# Fate of the first Brief Psychotic Disorder in hospitalised patients

**DOI:** 10.1192/j.eurpsy.2023.2248

**Published:** 2023-07-19

**Authors:** I. Bouguerra, W. Homri, M. Lagha, I. Ben Romdhan, N. Bram, R. Labben

**Affiliations:** 1 Razi Hospital, Mannouba, Tunisia; 2 C pyshciatry departement, Razi Hospital, Mannouba, Tunisia

## Abstract

**Introduction:**

Brief Psychotic Disorder (BPD), defined according to the DSM-5 by the presence of delusions and/or hallucinations and/or disorganised speech persisting for at least one day and less than one month, the disturbance not being due to a bipolar disorder or a schizophrenia spectrum disorder or to the effect of a substance. Classically, the prognosis of a BPD is considered to be divided between restitution ad integrum (30%), progression to bipolar disorder (30%), progression to schizophrenia (30%) or repetition of the same form (10%).

**Objectives:**

The objectives of our study were to evaluate the evolutionary modalities after the first hospitalization for BPD after a follow-up of at least one year and to compare them with the data in the literature.

**Methods:**

Our study was retrospective and descriptive. We reviewed the records of patients hospitalised in our department from 1 January 2014 to 31 December 2018 for a first BPD and assessed the subsequent course over a minimum period of one year.

**Results:**

We included 70 records of patients hospitalized. Twenty-five patients (35.71%) were lost to follow-up after their first hospitalisation. The remaining patients (64.29%) were divided into 3 groups according to the above-mentioned evolutionary modalities (recovery, recurrence of BPD, progression to schizophrenia, progression to bipolarity). Results were in favour of an evolution towards bipolar disorder (35.55%), towards schizophrenia (44.44%), a relapse of the BPD (4.44%), while 13.33% of the BPDs had no future after an aftercare of at least one year. In addition, one case of evolution towards a chronic delusional disorder of the persecution type was observed.

**Conclusions:**

In the present study, our results tend to be in line with the law of one-third described by some authors despite a slight discrepancy partly explained by the limitations of our study. Although, the outcome of BPD remains unpredictable. The minimum five years of evolution are decisive in assessing the subsequent prognosis.

**Disclosure of Interest:**

None Declared

